# The association analysis of *hOGG1* genetic variants and gastric cancer risk in a Chinese population

**DOI:** 10.18632/oncotarget.11802

**Published:** 2016-09-01

**Authors:** Jiafei Lu, Yongmei Yin, Mulong Du, Gaoxiang Ma, Yuqiu Ge, Qiang Zhang, Haiyan Chu, Na Tong, Meilin Wang, Jinrong Qiu, Zhengdong Zhang

**Affiliations:** ^1^ Department of Environmental Genomics, Jiangsu Key Laboratory of Cancer Biomarkers, Prevention and Treatment, Collaborative Innovation Center for Cancer Personalized Medicine, Nanjing Medical University, Nanjing, China; ^2^ Department of Genetic Toxicology, The Key Laboratory of Modern Toxicology of Ministry of Education, School of Public Health, Nanjing Medical University, Nanjing, China; ^3^ Department of Oncology, The First Affiliated Hospital of Nanjing Medical University, Nanjing, China; ^4^ Department of Biological-Therapy, Eastern Hepatobiliary Surgery Hospital Affiliated to Second Military Medical University, Shanghai, China

**Keywords:** polymorphism, hOGG1, gastric cancer, susceptibility

## Abstract

Human 8-oxoguanine DNA glycosylase (*hOGG1*) is known to play an important role in the prevention of carcinogenesis, including gastric cancer (GC). We performed a case-control study to investigate whether single nucleotide polymorphisms (SNPs) of *hOGG1* are associated with GC risk in a Chinese population. Two potential functional tagSNPs (rs159153 and rs1052133) and a previously reported risk SNP (rs125701) were genotyped in 1,275 GC patients and 1,436 controls. We found that SNP rs125701 G > A was significantly associated with the increased GC risk [adjusted odds ratio (OR) = 1.38, 95% confidence interval (CI) = 1.05-1.79 in additive model]. Besides, the functional studies demonstrated that the minor A allele of rs125701 significantly reduced the transcriptional activity of *hOGG1* promoter and enhanced the methylation level of CpG site of cg15357639. In conclusion, our results suggested that the SNP rs125701 in *hOGG1* promoter was associated with the elevated GC risk, which could act as a new potential biomarker for GC susceptibility. Further functional verification of rs125701 in GC pathogenesis is warranted.

## INTRODUCTION

Gastric cancer is the fifth most common malignancy worldwide, with an estimated 951,600 new cases and 723,100 deaths occurring in 2012 [1]. Although the decline trends in the GC morbidity have been noted in recent years, it remains the second leading cause of cancer deaths in China, with probably 498,000 deaths in 2015 [2]. The overall 5-year survival rate of GC is still poor, as GC patients are mostly diagnosed at advanced stage, during which any treatment is unreliable. Therefore, the identification and control of risk factors might be useful for reducing the prevalence of GC [3–5]. It is well-known that the pathogenetic mechanism of GC is very complicated. Accumulating evidence has proved that there exist a correlation between genetic polymorphisms and GC risk [6–8].

DNA damage is involved in carcinogenesis [9]. As a key component of DNA repair pathway, *hOGG1* encodes a DNA glycosylase specifically involved in the repair of DNA oxidative damage [10, 11]. The dysfunction of *hOGG1* might cause the DNA repair deficiency and then induce gene mutation and cell canceration. Abnormal expression of *hOGG1* was detected in several tumor tissues, such as ovarian cancer [12], breast cancer [13], and gastric cancer [14]. In addition, studies indicated that esophageal squamous cell carcinoma (ESCC) cells transfected with vector containing *hOGG1* could exhibit lower cell apoptosis, less oxidative damage, and longer survival ability compared with no-treated ESCC cells [15].

SNPs in DNA repair genes may conclusively affect individual variation in DNA repair capability and modulate individual cancer susceptibility. So investigation of SNPs in *hOGG1* contributes to uncovering pathogenesis of GC. Currently, the most frequently studied SNP of *hOGG1* was Ser326Cys polymorphism (rs1052133), still having inconsistent results with GC susceptibility [16–19]. In our previous study, we have conducted a meta-analysis to prove that the Ser326Cys polymorphism is significantly associated with an elevated risk of GC [20]. However, few studies have given attention to other *hOGG1* polymorphisms and GC susceptibility. In view of the importance of *hOGG1* in tumorigenesis, we thought that the roles of other SNPs in *hOGG1* deserve to be explored as well. Thus, in this study, we screened potential functional tagSNPs in *hOGG1* and its upstream 2000 bp region to explore the association between the SNPs in *hOGG1* and GC risk. In addition, SNPs in *hOGG1* that have been previously reported to be associated with cancers were recruited into our study.

## RESULTS

### Characteristics of study subjects

In this study, we found no significant difference among cases and controls in the distributions of age (*P* = 0.595) and sex (*P* = 0.349). Clinicopathological characteristics of GC patients are summarized in Supplementary Table S1. Of the cases, 61.3% of them were in non-cardia type, and 33.6% in the cardia and 5.1% in the both. In addition, 682 (61.4%) had lymph node metastasis and 167 (15.1%) existed distant metastasis. According to the TNM classification, all the cases were identified to stage I, II, III, and IV with the percentage of 23.1%, 24.6%, 35.5%, and 16.8%, respectively.

### Association analysis between the selected SNPs in *hOGG1* and GC risk

**Figure 1:**
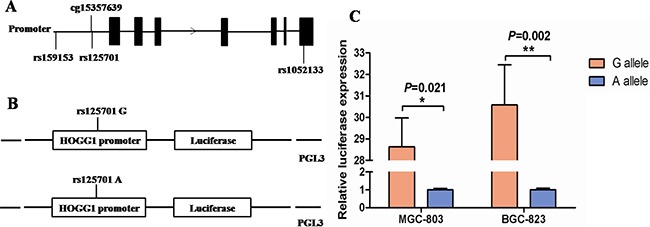
Effect of rs125701 polymorphism in the *hOGG1* promoter activity **A.** Relative position of selected three SNPs and a CpG site of cg15357639 in *hOGG1* gene. **B.** Schematic representation of reporter plasmids containing the rs125701 G or A allele, which was inserted upstream of the luciferase reporter gene in the pGL3 basic plasmid. **C.** The transcriptional activity of the constructs with rs125701 A allele was significantly lower than that of G allele in both MGC-803 and BGC-823 cells (*P* = 0.021 and 0.002, respectively). Columns represent mean from three independent experiments and bars means SD.

The position of three selected SNPs in *hOGG1* was shown in Figure 1A. The genotype distribution of the selected SNPs and their associations with GC risk are shown in Supplementary Table S2. The genotype frequencies of all SNPs among the controls were consistent with Hardy-Weinberg equilibrium (*P* = 0.260 for rs1052133, *P* = 0.125 for rs159153 and *P* = 0.518 for rs125701). We found that only SNP rs125701 showed significant difference between the cases and controls (*P*= 0.018 in adjusted additive model).

Subsequently, various inheritance models were used to determine the associations of rs125701 G>A polymorphism and GC risk (Table 1). Results of additive model indicated that the genotype distribution of rs125701 was significantly different between the cases and controls (adjusted OR = 1.38, 95% CI = 1.05-1.79, *P* = 0.018). Compared with the major GG genotype in a codominant model, the variant A alleles correlated to an increased risk of GC, showing a distinct allele-dosage effect (*P*_trend_ = 0.022). We found that SNP rs125701 also had risk effects on GC risk in the dominant model (adjusted OR = 1.33, 95% CI = 1.00-1.75, *P* = 0.047) and recessive model (adjusted OR = 9.14, 95% CI = 1.14-73.06, *P* = 0.037). Taken together, the rs125701 A allele was considered to be a potential risk allele for GC.

**Table 1:** Association of *hOGG1* rs125701 polymorphism with gastric cancer risk

Genotype	cases		controls		Adjusted OR(95%CI)^a^	*P*^a^
	N	%	N	%		
Codominant model						
GG	1159	90.9	1334	92.9	1.00(reference)	
AG	108	8.5	101	7.0	1.25(0.94,1.65)	0.127
AA	8	0.6	1	0.1	**9.29(1.16,74.23)**	**0.036**
*P* trend						**0.022**
Additive model					**1.38(1.05,1.79)**	**0.018**
Dominant model						
GG	1159	90.9	1334	92.9	1.00(reference)	
AG/AA	116	9.1	102	7.1	**1.33(1.00,1.75)**	**0.047**
Recessive model						
GG/AG	1276	99.4	1435	99.9	1.00(reference)	
AA	8	0.6	1	0.1	**9.14(1.14,73.06)**	**0.037**
Allele^b^						
G	2426	95.1	2769	96.4	1.00(reference)	
A	124	4.9	103	3.6	**1.37(1.05,1.80)**	**0.019**

a Adjusted by age, sex in logistic regression analysis

b Two-sided χ^2^ test for allele frequencies between the cases and controls

### Stratified analysis of SNP rs125701 and GC risk

We further evaluated the effects of rs125701 polymorphism on GC risk stratified according to demographic and clinicopathological characteristics. We did not find any association between rs125701 genotypes and GC susceptibility in subgroups of different age or sex (Supplementary Table S3). However, significant risk effect of rs125701 AG/AA genotype was observed among patients with non-cardia (adjusted OR = 1.53, 95% CI = 1.12-2.09), histological types of diffuse (adjusted OR = 1.43, 95% CI = 1.02-1.99) and lymph node metastasis (adjusted OR = 1.44, 95% CI = 1.04-1.99) (Table 2).

**Table 2:** Associations between rs125701 genotypes and clinical characteristics of GC

Variables	GG		AG/AA		Adjusted OR (95% CI)^a^	*P*^a^
	N	%	N	%		
Controls	1334	92.90	102	7.10	1.00(reference)	
Tumor site						
Cardia	373	92.56	30	7.44	1.08(0.70,1.65)	0.734
Non-cardia	658	89.65	76	10.35	**1.53(1.12,2.09)**	**0.008**
Histological types						
Diffuse	552	90.20	60	9.80	**1.43(1.02,1.99)**	**0.038**
Intestinal	470	91.62	43	8.38	1.22(0.94,1.77)	0.303
Depth of invasion						
T1	157	92.35	13	7.65	1.08(0.59,1.97)	0.798
T2	151	89.35	18	10.65	1.59(0.99,2.69)	0.088
T3	521	90.61	54	9.39	1.37(0.97,1.93)	0.075
T4	187	92.57	15	7.43	1.05(0.60,1.85)	0.856
Lymph node metastasis						
N0	397	92.76	31	7.24	1.03(0.68,1.56)	0.893
N1/N2/N3	615	90.18	67	9.82	**1.44(1.04,1.99)**	**0.028**
Distant metastasis						
M0	858	91.18	83	8.82	1.27(0.94,1.71)	0.127
M1	154	92.22	13	7.78	1.14(0.63,2.09)	0.662
TNM stages						
Localized (I+II)	499	90.89	50	9.11	1.33(0.93,1.90)	0.114
Advanced(III+IV)	550	91.06	54	8.94	1.30(0.92,1.83)	0.142

a Adjusted by age and sex in logistic regression analysis

### Effects of SNP rs125701 on transcriptional activity

To examine the biological effect of SNP rs125701 on the *hOGG1* promoter, different recombinant plasmids containing the *hOGG1* promoter region with rs125701 A or G allele (pGL3-GG/AA, Figure 1B) were transfected into MGC-803 and BGC-823 GC cells respectively. Then the relative transcriptional activity was evaluated via measuring firefly and renilla fluorescent intensity. As shown in Figure 1C, the luciferase activity of the vectors with rs125701 A allele was significantly decreased compared to that of G allele in both above cells (*P* = 0.021 and 0.002, respectively). These results indicated that rs125701 significantly affected the luciferase gene expression in vitro.

### In silico analysis for the allele-specific effect of rs125701

Next, the effect of SNP rs125701 on *hOGG1* expression was explored in the expression quantitative trait locus (eQTL) analysis through The Cancer Genome Atlas (TCGA) database. Regrettably, we did not discover any statistical difference of the *hOGG1* mRNA levels between different rs125701 genotypes from both GC patients and cancer-free controls (*P* = 0.541 for cases and 0.317 for controls) (Supplementary Figure S1).

**Figure 2:**
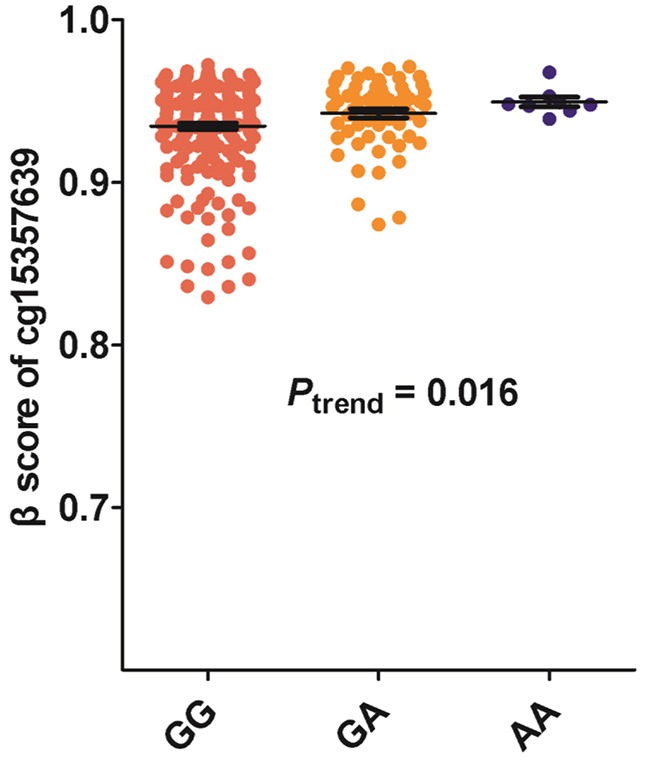
Association between rs125701 polymorphism and the methylation level of CpG site of cg15357639 There was prominent trend of increased methylation activity of CpG site of cg15357639 among the three genotype groups (*P*_trend_ = 0.016).

We also tested the CpG sites methylation status situated near the SNP rs125701 through UCSC database. Interestingly, we found a CpG site of cg15357639 with high methylation activity located 34 bases upstream of SNP rs125701 (Figure 1A). Thus we speculated whether the rs125701 polymorphism correlates to the methylation level of the CpG site and the methylation quantitative trait locus (meQTL) analysis was performed. As shown in Figure 2, prominent trend among the three genotype groups was existent: the more A allele, the higher methlation level of cg15357639 was found in GC patients (*P*_trend_ = 0.016).

## DISCUSSION

The *hOGG1* gene is located on chromosome 3p25-26, in which a frequent loss of heterozygosity was observed during tumorigenesis [21, 22]. Mice lacking *hOGG1* exhibit higher incidence of lung carcinogenesis and insulin resistance [23]. The abnormal expression of *hOGG1* was detected in several tumors [12, 13, 15] including GC [14]. In addition, the SNP rs1052133 in *hOGG1* and cancer susceptibility has been identified in numerous tumors including esophageal cancer [24], prostate cancer [25], lung cancer [26], breast cancer [27] and gastric cancer [28] and so on. All of these indicated important biological role of *hOGG1* in cancer etiology.

In this study, we conducted a case-control study to estimate the association between three candidate SNPs in *hOGG1* and GC risk. We found that individuals carrying the rs125701 AG/AA genotypes had a significantly increased GC risk, compared with those with the GG genotype. The SNP rs125701 was previously identified to have a suggestive association with high-grade prostate cancer in the US research [29] and a significantly reduced risk to bladder cancer from the Spanish Bladder Cancer Study (SBCS) [30]. In addition, the risk effect of rs125701 was more prominent among patients with non-cardia cancer than that with cardia cancer. Gastric cardia cancer differs from non-cardia cancer in anatomic site, etiology and clinical characteristics. Accumulating evidence has proved that many SNPs show different associations between the two main subsites of GC. SNP rs2294693 near *UNC5CL* at 6p21.1 [31] and SNP rs9841504 in *ZBTB20* [32] was reported to be associated with gastric non-cardia cancer. SNP rs2274223 located in *PLCE1* was associated with cardia but not non-cardia GC [33]. These findings that identified phenotype-specific genetic susceptibility loci may contribute to understanding the etiology and mechanisms of different subtypes of GC. The data published to date about GC risk and rs1052133 polymorphism is inconsistent. In our study, the SNP rs1052133 did not show a significant correlation to GC risk. This difference in susceptibility of rs1052133 to GC might be due to the different size of study sample or the gene-environment interactions and genetic background in different populations.

Currently, no functional study was performed to estimate the role of rs125701 polymorphism in the etiology of malignancy. In this study, luciferase reporter gene assay indicated that rs125701 A allele dramatically reduced the transcription activity of *hOGG1* promoter; and the rs125701 A allele was associated with elevated methylation level of CpG site of cg15357639 in the meQTL analysis. The rs125701 polymorphism is located adjacent to the CpG site of cg15357639, which is able to undergo methylation. Previous studies have demonstrated that DNA methylation is associated with SNPs, which may modify the CpG sites methylation or influence the generation of new CpG sites, change the status of genes' methylation and contribute to tumorigenesis in turn [34–36]. Furthermore, Juliet*et al.* found that functional risk SNPs (rs554219 and rs78540526) at the 11q13 locus for breast cancer could regulate *CCND1* expression through long-range regulation, which located approximately 125kb downstream [37]. Taken together, we speculated that the SNP rs125701 could suppress the expression level of other nearby genes instead of *hOGG1* by decreasing transcription activity or enhancing methylation level of the region where this SNP is located.

Several limitations of our study should be mentioned. First, as vital roles in gastric carcinogenesis, the smoking, drinking and *Helicobacter pylori* infection information were devoid in our study. The significance of rs125701 should be further validated to investigate the gene-environment interaction in the pathogenesis of GC. Second, the SNP rs125701 in additive model was of marginal difference between cases and controls after the Bonferroni correction (*P*= 0.054). The weak association between SNP rs125701 and GC risk might due to the small sample size in our study. This result should be verified by increasing the sample size or performing another case-control study in an independent population. Third, another six tagSNPs we identified in intron of *hOGG1* were neglected in our study, which may also be significant and their effect on GC risk should be evaluated.

In summary, we demonstrated that SNP rs125701 of *hOGG1* was associated with increased GC risk in the Chinese populations. Meanwhile, the polymorphism rs125701 leads to reduced transcription activity and enhanced methylation level of the promoter region, which might inhibit the expression of other nearby genes instead of *hOGG1*. These findings should be verified by larger, well-designed epidemiologic and functional studies.

## MATERIALS AND METHODS

### Study subjects

There were 1,275 GC cases and 1,436 cancer-free controls included in our study. All cases were histopathologically confirmed as gastric adenocarcinoma and recruited from the Cancer Clinical Research Base of Nanjing Medical University between March 2006 and May 2013. The control subjects were randomly enrolled at the same period when they sought physical examinations at hospital. In addition, the finally selected controls were frequency-matched to cases on age (±5 years) and sex. All eligible but only genetically unrelated ethnic Han Chinese patients were remained in this study. We acquired demographic and clinical information for all the subjects after a written informed consent was signed. A 5 mL peripheral venous blood sample was donated from each individual after the interview. The study was approved by the institutional review board of Nanjing Medical University.

### SNPs selection

SNPs located in *hOGG1* and its upstream 2000 bps region were searched based on genotype data of Asian population from the 1000 Genomes Project. We identified eight tagSNPs covering all the common SNPs (minor allele frequency, MAF > 0.05) using the Haploview 4.2 software (Cambridge, MA, USA) with a standard of *r*^2^ at least 0.8. We would like to focus on SNPs in functional region, including the region of promoter, 5′ -untranslated region (5′-UTR), and exon. Among the 8 tagSNPs, 2 of them are located in promoter (rs159153) and exon (rs1052133). Another 6 tagSNPs and their highly linked loci are all located in intron of *hOGG1* (Supplementary Table S4). Besides, SNPs that have been previously reported to be involved in cancer were recruited in our study. Finally, three SNPs (rs159153, rs1052133 and rs125701) remained in further analysis (Figure 1A).

### Genotyping

Genomic DNA was extracted from peripheral blood of each study subject. TaqMan allelic discrimination assay was used to genotype the selected SNPs by using the ABI 7900HT Real-Time PCR System (Applied Biosystems, Foster City, CA, USA). Sequences of primers and probes are summarized in Supplementary Table S5.

The average call rate of the three SNPs reached 99%. To control the quality, we randomly selected 10% of the samples to genotype again, and the results were 100% concordant.

### Construction of promoter reporter plasmids

As shown in Figure 1B, the *hOGG1* promoter region containing SNP rs125701 G or A allele was synthesized and then inserted into a pGL3-basic luciferase reporter plasmid (Promega, Madison, WI, USA) using the *Nhe*I and *Xho*I enzymes. The recombinant plasmids were sequenced to confirm the orientation and integrity of each construct.

### Transfection and luciferase assay

The luciferase reporter assay was used to detect the effect of SNP rs125701 on *hOGG1* promoter activity. MGC-803 and BGC-823 cells were cultured in 24-well plates and transfected with 800 ng of recombinant plasmids using Lipofectamine™ 2000 (Invitrogen, Carlsbad, CA, USA). The 10 ng Renilla luciferase pRL-SV40 (Promega, Madison, WI, USA) was simultaneously cotransfected into cells per well as internal control. After 24 hours transfection, the cells were lysed and measured by the Dual-Luciferase Reporter Assay System (Promega, Madison, WI, USA). Relative luciferase activity was estimated by the ratio of Firefly and Renilla fluorescent intensity. Each transfection was carried out in independent triplicate.

### Statistical analysis

The data were analyzed using SAS software (version 9.1.3; SAS Institute, Cary, NC). Student's *t* test and Pearson's chi-squared (χ^2^) test were used to assess the differences in demographic factors between cases and controls. Hardy-Weinberg equilibrium (HWE) of the controls was done by a goodness-of-fit χ^2^ test. The ORs and 95% CIs were calculated by an unconditional logistic regression model for the associations between genotype distribution and GC susceptibility. Variables of age and sex were as covariates adjusted for the association analysis. Multiple inheritance models were applied to estimate the significance of SNP rs125701. The promoter activity was analyzed by a Student's *t* test. *P* < 0.05 for two-sided was considered statistically significant.

## SUPPLEMENTARY MATERIALS FIGURE AND TABLES


