# Supplementary material for: The association analysis of hOGG1 genetic variants and gastric cancer risk in a Chinese population
Source: Oncotarget. 2016 Sep 1;7(40):66061–8. doi: 10.18632/oncotarget.11802 (PMC5323214; doi:10.18632/oncotarget.11802)
Supplement: Supplementary file 1 [file oncotarget-07-66061-s001.pdf]

The association analysis of *hOGG1* genetic variants and gastric cancer risk in a Chinese population

SUPPLEMENTARY FIGURE AND TABLES

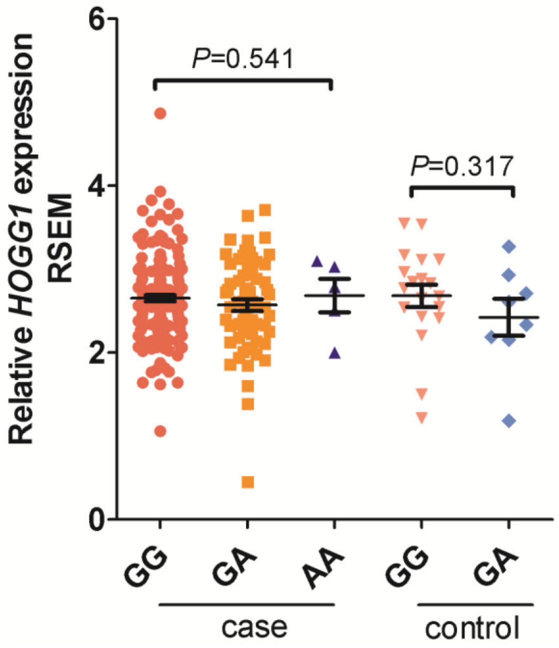

Supplementary Figure S1: Association between rs125701 polymorphism and the expression levels of *hOGG1* mRNA from The Cancer Genome Atlas database. There was no significant difference between SNP rs125701 and mRNA expression of *hOGG1* in both gastric cancer patients and cancer-free controls (P = 0.541 and 0.371, respectively).

Supplementary Table S1: Frequency distributions of selected variables between the gastric cancer cases and controls

Variables	Cases (n=1275)		Controls (n=1436)		P^a
	N	%	N	%	
Age (years) (mean±SD)	63.1±10.7		63.3±11.0		0.595
Sex					
Male	879	69.2	969	67.5	0.349
Female	392	30.8	467	32.5	
NA	4				
Tumor sites					
Cardia	403	33.6			
Non-cardia	734	61.3			
Both	61	5.1			
NA	77				
Histological types					
Diffuse	612	54.4			
Intestinal	513	45.6			
NA	150				
Depth of invasion					
Tis	1	0.1			
T1	170	15.2			
T2	169	15.1			
T3	575	51.5			
T4	202	18.1			
NA	158				
Lymph node metastasis					
N0	428	35.6			
N1/N2/N3	682	61.4			
NA	165				
Distant metastasis					
M0	941	84.9			
M1	167	15.1			
NA	167				
TNM stages					
I	267	23.1			
II	284	24.6			
III	410	35.5			
IV	194	16.8			
NA	120				

[a] Two-sided student t test for the frequency distributions of age between the cases and controls
Two-sided χ^2 test for the frequency distributions of sex between the cases and controls

Supplementary Table S2: The genotype frequencies and MAF between the gastric cancer cases and controls

SNPs	Allele[a]	Cases[b]	Controls[b]	MAF[c]		HWE[d]	*P*[e]
				Cases	Controls		
rs1052133(exon)	G/C	477/591/211	525/702/207	0.396	0.389	0.260	0.687
rs159153(promoter)	T/C	1030/229/16	1146/277/10	0.102	0.104	0.125	0.862
rs125701(promoter)	G/A	1159/108/8	1334/101/1	0.049	0.036	0.518	**0.018**

[a] major allele/minor allele of the three SNPs respectively
[b] major homozygote/heterozygote/minor homozygote of the genotypes between the cases and controls
[c] MAF, minor allele frequency in cases and controls
[d] HWE (Hardy–Weinberg equilibrium) test among controls
[e] Adjusted by age, sex in logistic regression analysis in additive model

Supplementary Table S3: Stratification analyses of demographic features between rs125701 genotype and GC risk

Variables	Genotypes (Cases/Controls)				Adjusted OR (95%CI)[a]	*P*[a]
	GG		AG/AA			
	N	%	N	%		
Total	1159/1334	90.90/92.90	116/102	9.1/7.1	1.33(1.00,1.75)	**0.047**
Age						
≤ 65	644/737	89.82/92.24	73/62	10.18/7.76	1.34(0.94,1.93)	0.109
> 65	507/597	92.18/93.72	43/40	7.82/6.28	1.33(0.85,2.11)	0.217
Sex						
Male	797/900	90.57/92.88	83/69	9.43/7.12	1.32(0.94,1.85)	0.107
Female	359/434	91.58/92.93	33/33	8.42/7.07	1.28(0.76,2.16)	0.351

[a]Adjusted by age or sex in logistic regression analysis

Supplementary Table S4: The selected tagSNPs and their captured SNPs in *hOGG1*

TagSNPs	SNPs captured by TagSNP	Position	Location in gene region	r^2 value
rs159153		9789875	promoter	
rs55814656		9795194	intron	
	rs3219008	9795543	intron	1.0
rs55841843		9795752	intron	
	rs73021447	9795910	intron	0.939
rs2075747		9797723	intron	
rs1052133		9798773	exon	
	rs2072668	9798140	intron	1.0
	rs4021704	9800444	intron	0.901
	rs73021455	9806657	intron	0.918
	rs6763347	9803796	intron	0.918
	rs2304277	9801080	intron	0.858
	rs7609858	9802630	intron	0.897
rs17252807		9803126	intron	
	rs7617500	9802599	intron	1.0
rs293794		9803627	intron	
rs146018083		9806884	intron	

Supplementary Table S5: Sequences of primers and probes for *hOGG1* SNPs

Genetic Variation	Name	Sequence (5'-3')
rs1052133	Primer-F	CCCTCCTACAGGTGCTGTTCA
	Primer-R	AACCCTTTCTGCGCTTTGCT
	FAM	CCAATGCCGCCATG
	HEX	CGCCAATCCCGCCA
rs159153	Primer-F	GGATTACGCAAAGCTGAATGTCT
	Primer-R	CAGCAAAGAGAGCCCCTGTT
	FAM	TGCTCAGCACAGGG
	HEX	AAGGTGCTCAACACA
rs125701	Primer-F	GGTCTCTGGCTGGGACACAA
	Primer-R	CCCAGACTGCAAATTCTTGAAGA
	FAM	CAATCCGCTGCAGAT
	HEX	TCAATCCACTGCAGATG
